# Host identification for the deep-sea snail genus *Haliella* with description of a new species (Caenogastropoda, Eulimidae)

**DOI:** 10.3897/zookeys.908.46613

**Published:** 2020-02-03

**Authors:** Tsuyoshi Takano, Shoichi Kimura, Yasunori Kano

**Affiliations:** 1 Meguro Parasitological Museum, 4-1-1 Shimomeguro, Meguro, Tokyo 153-0064, Japan Meguro Parasitological Museum Meguro Japan; 2 Department of Life Sciences, Graduate School of Bioresources, Mie University, 1577 Kurimamachiya, Tsu, Mie 514-8507, Japan Mie University Tsu Japan; 3 Atmosphere and Ocean Research Institute, The University of Tokyo, 5-1-5 Kashiwanoha, Kashiwa, Chiba 277-8564, Japan The University of Tokyo Chiba Japan

**Keywords:** *
Brissopsis
*, Echinoidea, Gastropoda, irregular sea urchin, parasite, Vanikoroidea

## Abstract

A new parasitic species of eulimid gastropod, *Haliella seisuimaruae***sp. nov.**, is described from bathyal (728–978 m) waters off the Pacific coasts of Japan. It shows the closest resemblance to the type species *H. stenostoma* from the North Atlantic and Barents Sea in having a tall shell with an almost straight outer lip, but differs in having a junction of the parietal wall and columellar lip at 38% of the aperture height from the suture (33% in *H. stenostoma*), a slightly wider aperture and a more curved and extended columellar lip. The holotype of *H. seisuimaruae***sp. nov.** was found attached to an irregular sea urchin, Brissopsis sp. cf. luzonica (Spatangoida: Brissidae). This represents the first direct observation of parasitic ecology and echinoderm host for the genus *Haliella*. A new replacement name, *Eulima tsushimensis***nom. nov.**, is proposed here for *Eulima stenostoma* A. Adams, 1861, which is preoccupied by *Eulima stenostoma* Jeffreys, 1858 (type of *Haliella*).

## Introduction

Snails of the family Eulimidae (Caenogastropoda: Vanikoroidea) are exclusive parasites of echinoderms including all five classes of the phylum, namely Asteroidea (sea stars), Crinoidea (sea lilies and feather stars), Echinoidea (sea urchins), Holothuroidea (sea cucumbers) and Ophiuroidea (brittle stars; Warén 1984). However, many eulimid species are temporary parasites and have therefore been collected only as free-living specimens. Moreover, deep-sea eulimids tend to be detached from the host when they are collected with bottom sediment and other benthos by such destructive gears as a biological dredge and a beam trawl (see Schiaparelli et al. 2007; Takano et al. 2018). These result in a lack of host information for a number of eulimid species and even genera (Warén 1984; Bouchet and Warén 1986; Hori and Matsuda 2017).

*Haliella* Monterosato, 1878 is one of such deep-sea genera of Eulimidae without host information. The type species *Haliella stenostoma* (Jeffreys, 1858) occurs over a wide depth range (50–3000 m) in the North Atlantic and Barents Sea (Warén 1984; Bouchet and Warén 1986; Nekhaev 2013). The shell of *H. stenostoma* is characterized by (1) a slender cylindrical outline, (2) a height of up to 12.5 mm, (3) a blunt apex, (4) slightly convex whorls and (5) a very high aperture with a strongly twisted columella (Jeffreys 1858; Bouchet and Warén 1986). Other diagnostic characters of the species include the lack of pigmented eyes and protandrous hermaphroditic sex determination (Warén 1984; Bouchet and Warén 1986). Bouchet and Warén (1986) have further suggested its early development as a non-planktotrophic larva that hatches at a shell height of 700 µm. Parasitic ecology, however, remains undocumented except that its slender shell and broad foot (Bouchet and Warén 1986) may imply the presence of a free-living period (see Warén 1984; Vermeij 1993).

Five more species have been described under this genus (MolluscaBase 2019). These inhabit shelf and/or bathyal depths in various parts of the world’s oceans. *Haliella abyssicola* Bartsch, 1917 and *H. chilensis* Bartsch, 1917 occur in the eastern Pacific, off California and Chile, respectively (Bartsch 1917); *H. canarica* Bouchet & Warén, 1986 in the northeastern Atlantic (off the Canary Islands; Bouchet and Warén 1986); *H. ventricosa* Feng, 1996 in the South China Sea (Feng 1996). *Haliella tyrrhena* Di Geronimo & La Perna, 1999 was originally described from fossil material in the Quaternary marine deposits of the Tyrrhenian Sea, Mediterranean (e.g., Di Geronimo and La Perna 1999; Di Geronimo et al. 2001; Romani et al. 2016), but the occurrence of fresh empty shells suggests this to be an extant species (Giacobbe and Notaristefano 2018). Moreover, several unnamed congeners have been reported from bathyal depths around Japan (Hasegawa 2001, 2005, 2009; Hasegawa and Okutani 2011). None of these named or unnamed species have previously been collected *in situ* on their hosts, whereas bycatch in the same trawl/dredge hauls suggests they might parasitize on brittle stars (Warén 1984) or irregular sea urchins (Warén in Hasegawa 2009).

**Figures 1–3.**
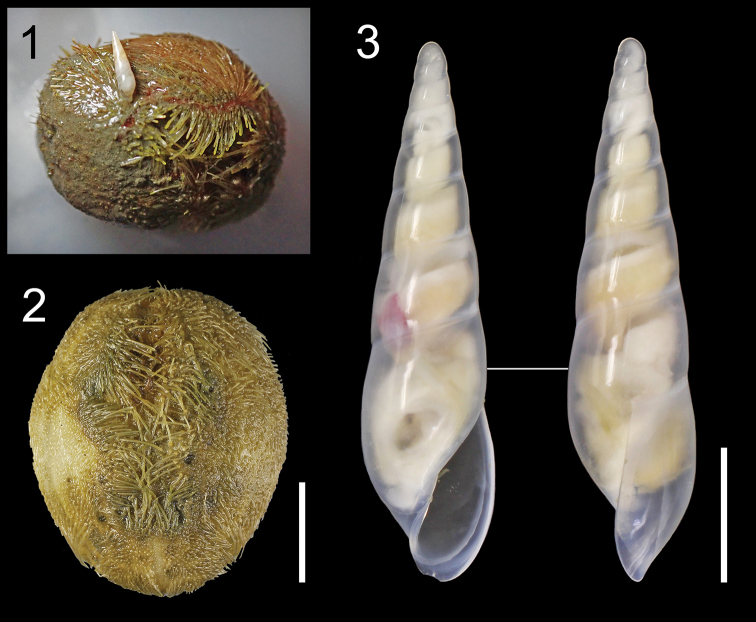
Holotype of *Haliella seisuimaruae* sp. nov. and its host sea urchin Brissopsis sp. cf. luzonica (both NSMT-Mo 79088). **1** Photograph of living holotype, attaching to latero-dorsal surface of sea urchin test **2** dorsal or aboral view of same host specimen **3** holotype, apertural and lateral views. Scale bars: 10 mm (**2**); 2 mm (**3**).

Here, we describe *Haliella seisuimaruae* sp. nov. from bathyal waters in Japan. The holotype of the species was found attached to an irregular sea urchin, Brissopsis sp. cf. luzonica (Gray, 1851) (Echinoidea: Spatangoida: Brissidae; Figs 1–3). This represents the first direct observation of parasitic ecology and echinoderm host for the genus *Haliella*.

## Materials and methods

The holotype of *Haliella seisuimaruae* sp. nov. (Fig. 3) was collected with a 2-m wide beam trawl from a muddy bottom at an depth of 641–727 m, off Tanabe, Wakayama, Honshu Island, Japan, on the No. 1803 research cruise of T/V “Seisui-maru” (station B2; Table 1). This specimen was found attached to the dorsal (aboral) side of an irregular sea urchin, Brissopsis sp. cf. luzonica (Fig. 1). The same trawl haul yielded hundreds of individuals of this sea urchin species, whereas no other specimens of *H. seisuimaruae* sp. nov. were found there, neither attached to nor detached from the host species. Both snail and host sea urchin were preserved in pure ethanol immediately after sampling and photo-documentation.

In regard to the identification of the host, *Brissopsis luzonica* was described very briefly from Luzon Island, Philippines, without accompanying illustration or depth information (Gray 1851). More recent specimens from the tropical Indo-West Pacific (Copperd 2008: figs 3, 4) show differences from the present host sea urchin (Fig. 2) and other Japanese material (e.g., Morishita 1969: pl. 25, figs 5–7; Jimi et al. 2018: fig. 3B) in test morphology, suggesting the presence of multiple species under the same name.

**Figures 4, 5.**
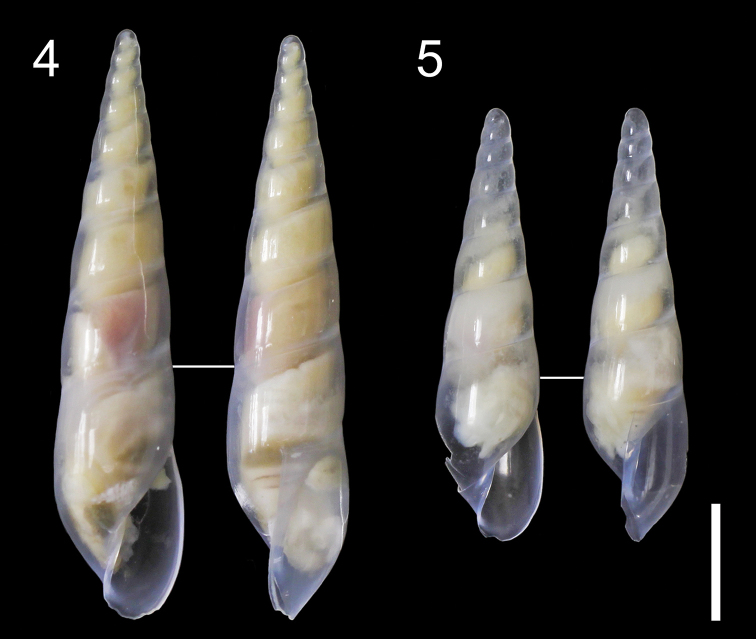
Paratypes of *Haliella seisuimaruae* sp. nov., apertural and lateral views. **4** Paratype 1 (MPM Coll. No. 21596) **5** paratype 2 (AORI YK1520). Scale bar: 2 mm.

Two paratypes of the new species (Figs 4, 5) were collected from bathyal waters of Tosa Basin, off Kochi, Shikoku Island, Japan, on the KT-11-12 cruise of R/V “Tansei-maru” (Table 1). Two trawl hauls at depths of 728–746 m and 955–978 m contained solitary individuals, both detached from a host. The type specimens and host sea urchin were deposited in the National Museum of Nature and Science, Tokyo (**NSMT**), Meguro Parasitological Museum (**MPM**), and Atmosphere and Ocean Research Institute, The University of Tokyo (**AORI**).

**Table 1.** Localities of type specimens of *Haliella seisuimaruae* sp. nov.

Specimen	Locality	Coordinates	Depth	Cruise, station number
Holotype	Off Tanabe, Wakayama, Japan	33°32.2'N, 135°08.1'E	641–727 m	T/V “Seisui-maru” No. 1803, B2
Paratype 1	Tosa Basin, off Kochi, Japan	33°00.7'–01.9'N, 133°45.8'–45.4'E	955–978 m	R/V “Tansei-maru” KT-11-12, K5
Paratype 2	Tosa Basin, off Kochi, Japan	33°05.5'–04.4'N, 134°03.4'–03.6'E	728–746 m	R/V “Tansei-maru” KT-11-12, K6-2

The snail and sea urchin specimens were sequenced for the mitochondrial cytochrome *c* oxidase subunit I (COI) gene to confirm their systematic positions and to provide molecular-based identification criteria. Total DNA was extracted from the foot of the three snail specimens, as well as the intestinal tissue of the host *Brissopsis*, using a DNeasy Blood and Tissue Kit (Qiagen). Partial fragments of the COI gene were amplified with Folmer et al.’s (1994) invertebrate-universal primers (LCO1490: GGTCAACAAATCATAAAGATATTGG and HCO2198: TAAACTTCAGGGTGACCAAAAAATCA) for the eulimid snails and Hoareau and Boissin’s (2010) echinoderm-specific primers (COIceF: ACTGCCCACGCCCTAGTAATGATATTTTTTATGGTNATGCC and COIceR: TCGTGTGTCTACGTCCATTCCTACTGTRAACATRTG) for the sea urchin. PCR amplification was conducted in a total volume of 25 µl: 17.5 µl DDW, 0.13 µl Ex *Taq* HS (TaKaRa Bio Inc.), 2.5 µl Ex *Taq* Buffer (10×), 2.0 µl dNTP mixture (2.5 mM each), 0.3 µl forward and reverse primers (20 µM each) and 2.5 µl DNA solution; see Takano and Kano (2014) and Takano et al. (2018) for details. Amplicons were purified with ExoSAP-IT (Applied Biosystems, ABI) following the described protocol and then sequenced using the amplification primers and a BigDye Terminator Cycle Sequence Kit v. 3.1 (ABI). After purification with a BigDye XTerminator Purification Kit (ABI), the reaction mixtures were analyzed on an ABI 3130xl sequencer at AORI.

Phylogenetic trees were reconstructed separately for the snail and host using the maximum likelihood (ML) method. Newly determined fragments of the COI gene were aligned in MEGA7 (Kumar et al. 2016) together with published sequences of vanikoroid snails or spatangoid sea urchins (see Stockley et al. 2005; Takano and Kano 2014). ML trees were reconstructed using RAxML v. 8.2.10 (Stamatakis 2014) with the GTR + G model. Nodal support was assessed with bootstrap resampling (1000 replicates). The obtained trees were edited in FigTree v. 1.4.3 (available at: http://tree.bio.ed.ac.uk/software/figtree/).

## Results

The holotype and paratype 2 of *Haliella seisuimaruae* sp. nov. yielded near-identical sequences of the COI gene (DDBJ/EMBL/GenBank accession numbers LC514118 and LC514119), with one missense and two silent nucleotide substitutions out of 633 base pairs (bp). No successful amplification was achieved for the paratype 1. Although the caenogastropod affinity of the sequences was verified through BLAST searches, ML tree reconstruction based on this fast-evolving gene resulted in almost no resolution among genera within the superfamily Vanikoroidea or the family Eulimidae (tree not shown). *Haliella seisuimaruae* sp. nov., the only representative of the genus in the tree, ambiguously clustered with *Vanikoro cancellata* (Lamarck, 1822) (KT149317) of the polyphyletic Vanikoridae with a bootstrap probability (BP) value of 24%.

**Figure 6.**
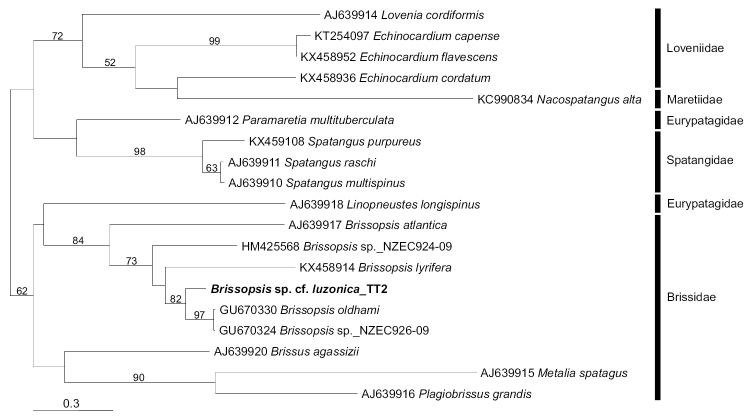
Maximum likelihood tree for host sea urchin (Brissopsis sp. cf. luzonica_TT2; NSMT-Mo 79088) and its relatives inferred from partial COI-gene sequences (655 bp). Numbers above branches denote bootstrap percentages (1000 replicates; values below 50% are not shown). DDBJ/EMBL/GenBank accession numbers are given for published sequences.

The sea urchin parasitized by the holotype of *H. seisuimaruae* sp. nov. (NSMT-Mo 79088; DNA_TT#2 in MPM) yielded a COI sequence that confirms its assignment to the genus *Brissopsis* Agassiz, 1840 (655 bp, LC514120). This sequence showed 94.5% similarity with the published COI sequences of *B. oldhami* Alcock, 1893 (e.g., GU670330) and *Brissopsis* sp. NZEC926-09 (GU670324) in the overlapping 490-bp region. The ML tree reconstruction for the spatangoid sea urchins recovered the species of *Brissopsis*, including the host of *H. seisuimaruae* sp. nov., as a monophyletic group (Fig. 6; BP: 84%). The host sea urchin formed the sister clade to *B. oldhami* + *Brissopsis* sp. NZEC926-09 (BP: 82%).

### Taxonomy

**Superfamily Vanikoroidea Gray, 1840**

**Family Eulimidae Philippi, 1853**

#### Genus Haliella

Taxon classificationAnimaliaLittorinimorphaEulimidae

Monterosato, 1878

F2ED913B-078B-5CFF-AA12-08EFCFE09EB3

##### Type species.

*Eulima stenostoma* Jeffreys, 1858, by monotypy.

#### Haliella seisuimaruae sp. nov.

Taxon classificationAnimaliaLittorinimorphaEulimidae

23C0F9F1-E62A-58EA-95C7-17425872C362

http://zoobank.org/FDCA45D1-C71D-47EE-9BE5-0946D12FEB50

Figs 1 , 3 , 4–5

##### Type material.

***Holotype*** (NSMT-Mo 79088) from the type locality, found attached to the test of Brissopsis sp. cf. luzonica (Figs 1, 2), collected on 28 April 2018 by S. Kimura. ***Paratype 1*** (MPM Coll. No. 21596) and ***paratype 2*** (AORI YK1520) from Tosa Basin, off Kochi, Shikoku Island, Japan without host information, both collected on 24 June 2011 by T. Takano, Y. Kano and H. Fukumori (Tables 1, 2).

**Table 2.** Voucher and shell measurements of type specimens of *Haliella seisuimaruae* sp. nov.

Specimen	Voucher	Preservation	DNA number	Accession number	Shell height	Shell width	Aperture height	Aperture width	Number of whorls
Holotype	NSMT-Mo 79088	Pure ethanol	AORI_YK#3469	LC514118	7.8 mm	2.0 mm	2.5 mm	1.2 mm	7.8
Paratype 1	MPM Coll. No. 21596	Dry	AORI_YK#1473	–	9.8 mm	2.2 mm	2.8 mm	1.3 mm	9.0
Paratype 2	AORI YK1520	Pure ethanol	AORI_YK#1520	LC514119	7.1 mm	1.8 mm	2.1 mm	1.1 mm	7.5

##### Type locality.

Off Tanabe, Wakayama, Honshu Island, Japan; 33°32.2'N, 135°08.1'E; 641–727 m deep.

##### Etymology.

The name refers to the T/V “Seisui-maru” of Mie University, which captured the holotype of the new species.

##### Distribution.

Known only from the localities of the type specimens.

##### Diagnosis.

A typical *Haliella* species with high, narrow aperture. Columellar lip tilted at 20° angle from coiling axis, twisted, extended outward near base; junction of parietal wall and columella at ca. 38% of aperture height from suture.

##### Description.

Shell slender, conical with blunt apex, up to 9.8 mm high, 2.2 mm wide and 9 slightly convex whorls (7.8 mm high, 2.0 mm wide, 7.8 whorls in holotype), smooth, thin but not fragile, translucent white. Protoconch large, smooth, paucispiral with 1.5 whorls, indistinctly demarcated from teleoconch. Teleoconch whorls bear straight, irregularly spaced incremental lines or growth pause scars, which are situated at 0.5, 1.6, 2.2, 2.9, 3.4, 3.7, 4.4, 4.7, 5.3 and 5.8 whorls in holotype. Body whorl occupies 45% of total shell height. Aperture high, narrow and oblique; outer lip simple, only slightly curved or almost straight in lateral view, with its most protruding part at 3/4 of aperture height from suture; parietal callus absent; columellar lip tilted at angle of 20° from coiling axis in holotype, thick, twisted, extended outward near round base; junction of parietal wall and columellar lip placed at ca. 38% of aperture height from suture. Umbilicus absent. Operculum teardrop shaped, thin, yellowish. Pigmented eyes absent.

##### Remarks.

The generic position of the present species is determined most readily by its shell with (1) a slender and cylindrical outline, (2) a blunt apex, (3) slightly convex whorls, and (4) a high, narrow aperture with (5) a twisted columella (Figs 3–5). In addition, (6) the absence of pigmented eyes agrees with the condition reported for the type species *H. stenostoma* from the North Atlantic and Barents Sea (Bouchet and Warén 1986). Actually, the present new species shows the closest resemblance to *H. stenostoma* in having a tall shell with an almost straight outer lip with its most protruding part at 3/4 of the aperture height from the suture. However, *H. seisuimaruae* sp. nov. has a junction of the parietal wall and columellar lip at 38% of the aperture height (33% in *H. stenostoma*), a slightly wider aperture and a more curved and extended columellar lip (Fig. 3; see Warén 1984; Nekhaev 2013 for comparison).

*Haliella seisuimaruae* sp. nov. is more easily distinguished from the five other described species of the genus. *Haliella ventricosa* from the South China Sea is characterized by a larger body whorl relative to the total shell height (> 60%) and an accordingly higher aperture (Feng 1996). *Haliella abyssicola* from off California differs in having a columellar lip that almost parallels to the coiling axis (Bartsch 1917). The other three congeners, *H. chilensis* from Chile, *H. canarica* from the northeastern Atlantic and *H. tyrrhena* from the Mediterranean, have a much wider, more ovate shell aperture (Bartsch 1917; Bouchet and Warén 1986; Di Geronimo and La Perna 1999).

Hasegawa (2001, 2005, 2009) and Hasegawa and Okutani (2011) reported four eulimid species from Japanese bathyal waters as undescribed members of *Haliella*. The shell outline and apertural features also distinguish *H. seisuimaruae* sp. nov. from these unnamed taxa. A species from off Miyako, Iwate, northern Honshu shares a near-straight outer lip with the present new species but differs in having a more tapering apex (Hasegawa 2009: figs 142, 143). The second species from Tosa Bay, Shikoku Island and the third from the East China Sea have the parietal wall and columellar lip joining in a straight line (Hasegawa 2001: pl. 2, fig. L; Hasegawa 2005: fig. 7H). The fourth species from Sagami Bay shows a strongly curved outer lip with its protruding part at half of the total aperture height (Hasegawa and Okutani 2011: fig. 20).

As a side note, Adams (1861: 126) described *Eulima stenostoma* A. Adams, 1861 from “26 fathoms, Tsu-Sima” (Tsushima, Japan), unaware of the primary homonym *Eulima stenostoma* Jeffreys, 1858 (now the type species of *Haliella*). Identical species-group names established for different nominal taxa when originally combined with the same generic name are primary homonyms and the junior name is permanently invalid (International Code of Zoological Nomenclature, Article 57.2). The holotype of the Adams’s species (BMNH 1878.1.28.127 in the Natural History Museum, London; see Higo et al. 2001: 57, fig. G2044) most closely resembles the aforementioned, alleged *Haliella* from Sagami Bay (Hasegawa and Okutani 2011) in the apertural morphology of the shell. This latter species from Sagami Bay, however, is actually a member of the ophiuroid-parasite genus *Eulima* (T. Takano, unpublished data), as probably is the case with Adams’s *stenostoma*. In order to prevent further confusion between species and genera, we propose a new substitute name, *Eulima tsushimensis* Takano, Kimura & Kano nom. nov., for *E. stenostoma* A. Adams, 1861 with Adams’s original specimen as the holotype.

This study presents the first direct observation of parasitic ecology and echinoderm host for the genus *Haliella*. The holotype of *H. seisuimaruae* sp. nov. was found attached to the dorsal (aboral) side of an irregular sea urchin, Brissopsis sp. cf. cf. luzonica (Fig. 1). Eulimid species in a single genus generally exploit hosts of the same echinoderm class (Warén 1984), suggesting that the other congeners may also parasitize (irregular) sea urchins. Previously known parasites of irregular sea urchins include the species of three other eulimid genera, *Clypeastericola* Warén, 1994, *Hypermastus* Pilsbry, 1899 and *Turveria* Berry, 1956 (Warén and Crossland 1991; Warén et al. 1994; Matsuda et al. 2010, 2013a, b). Those eulimids, however, appear to be distantly related to *Haliella* with rather different shell morphology (see Warén 1984). They inhabit much shallower waters ranging from intertidal to shallow subtidal zones, with *Hypermastus bulbulus* (Murdoch & Suter, 1906) from 200 m deep as a single known exception (Warén and Crossland 1991; Warén et al. 1994). The host specialization to irregular sea urchins might thus have occurred more than once in the evolutionary history of the Eulimidae.

### Key to species of *Haliella* Monterosato, 1878

**Table:** 

1	Body whorl occupies ≥ 0.6 times of total shell height	***H. ventricosa* Feng, 1996**
–	Body whorl occupies < 0.6 times of total shell height	**2**
2	Shell aperture ≥ 2 times as high as wide	**3**
–	Shell aperture < 2 times as high as wide	**5**
3	Columellar lip almost parallel to coiling axis	***H. abyssicola* Bartsch, 1917**
–	Columellar lip oblique to coiling axis	**4**
4	Junction of parietal wall and columellar lip at 33% of aperture height from suture	***H. stenostoma* (Jeffreys, 1858)**
–	Junction of parietal wall and columellar lip at 38% of aperture height from suture	***H. seisuimaruae* sp. nov.**
5	Parietal wall and columellar lip join in a straight line	***H. canarica* Bouchet & Warén, 1986**
–	Junction of parietal wall and columellar lip distinct	**6**
6	Shell small, ≤ 3 mm high with up to ca. 5 whorls	***H. tyrrhena* Di Geronimo & La Perna, 1999**
–	Shell large, > 5 mm high with more than 7 whorls	***H. chilensis* Bartsch, 1917**

## Supplementary Material

XML Treatment for Haliella

XML Treatment for Haliella seisuimaruae
